# Hybrid bronchoscopic and surgical resection of endotracheal angiomatoid fibrous histiocytoma

**DOI:** 10.1186/s13019-019-0861-7

**Published:** 2019-02-28

**Authors:** Wobbe Bouma, Kor Johan Koning, Albert J. H. Suurmeijer, Dirk Jan Slebos, Massimo A. Mariani, Theo J. Klinkenberg

**Affiliations:** 1 Department of Cardiothoracic Surgery, University of Groningen, University Medical Center Groningen, Groningen, The Netherlands; 2 Department of Pulmonary Diseases, University of Groningen, University Medical Center Groningen, Groningen, The Netherlands; 3 Department of Pathology, University of Groningen, University Medical Center Groningen, Groningen, The Netherlands

**Keywords:** Angiomatoid fibrous histiocytoma, Bronchoscopy, Tracheal resection

## Abstract

**Background:**

Angiomatoid fibrous histiocytoma (AFH) is a soft-tissue tumor that generally affects the extremities of children and young adults. AFH overlaps with primary pulmonary myxoid sarcoma (PPMS) and can occur in unusual locations.

**Case presentation:**

We present a case of a 22-year-old female with AFH in the distal trachea. In addition to describing the challenge in making a correct diagnosis of AFH, we describe the first case of successful hybrid bronchoscopic and surgical resection of endotracheal AFH. A staged removal procedure was required to quickly secure the airway, allowing a lower-risk elective distal tracheal resection through a cervical approach for complete resection. A more conventional, but more invasive, more painful and cosmetically less satisfying thoracotomy was avoided.

**Conclusions:**

A distal tracheal resection for AFH can be safely performed in young adults through a cervical approach with excellent follow-up results.

## Background

Angiomatoid fibrous histiocytoma (AFH) is a soft-tissue tumor that generally affects the extremities of children and young adults, but occasionally occurs at unusual locations such as the trachea [1–3]. This case-report demonstrates the challenge in making a correct (histological) diagnosis of endotracheal AFH and presents the first case of successful hybrid bronchoscopic and surgical resection of endotracheal AFH.

## Case presentation

A 22-year-old female was referred with dyspnea and wheezing and an initial diagnosis of allergic asthma. Several weeks before she was admitted to the intensive care unit with acute respiratory failure due to a presumed severe asthma exacerbation. After weaning from mechanical ventilation she received formoterol and beclomethasone. Auscultation revealed pulmonary wheezing and a high-pitched stridor. Spirometry showed expiratory airflow obstruction and signs of severe fixed intrathoracic stenosis.

**Fig. 1:**
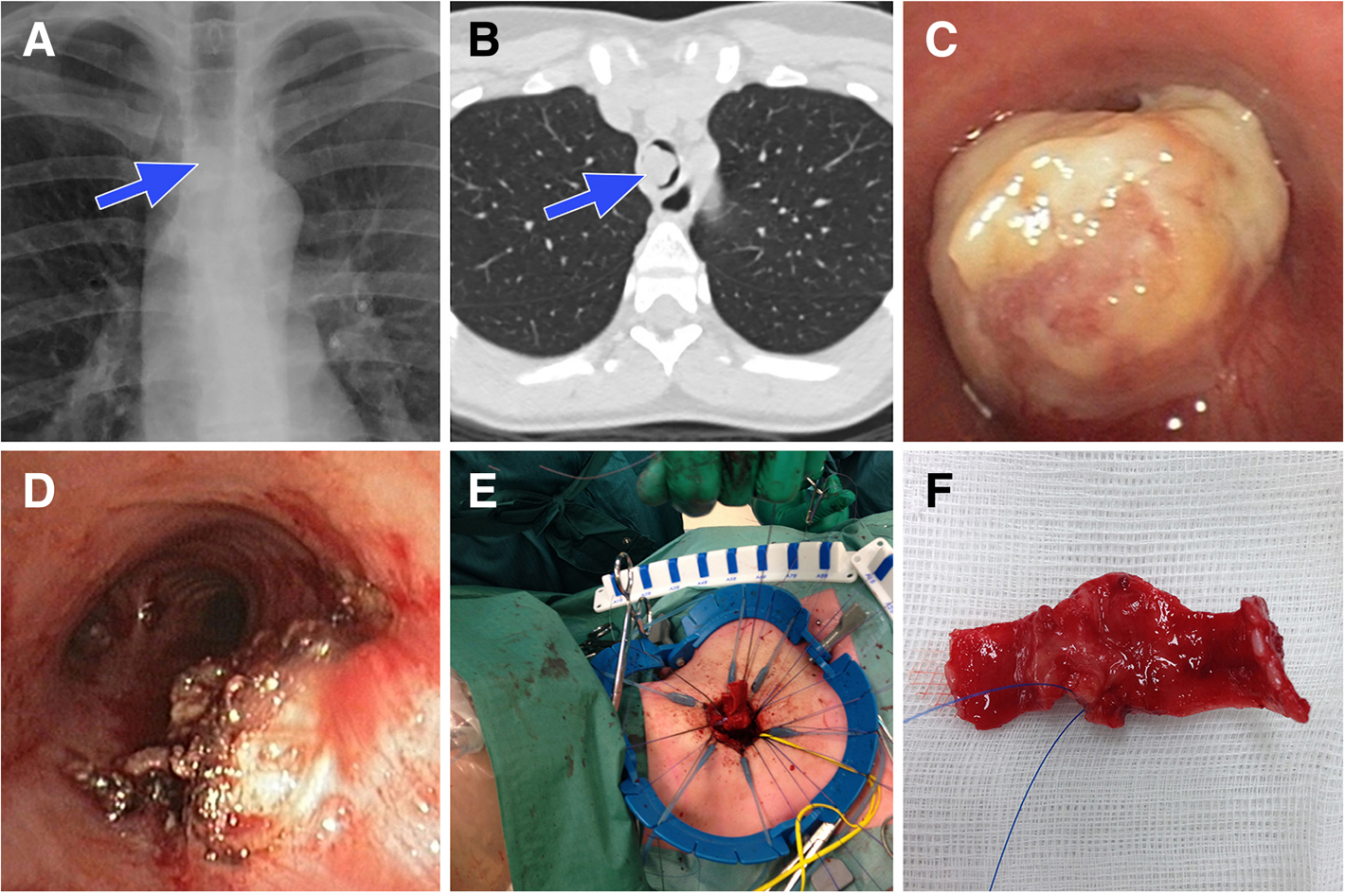
Endotracheal angiomatoid fibrous histiocytoma imaging and resection

In retrospect, previous chest X-rays showed an intratracheal mass close to the carina (Fig. 1a, blue arrow). Emergency computed tomography (CT) confirmed the presence of a large obstructing intratracheal mass (Fig. 1b, blue arrow). Emergency bronchoscopy was performed under general anesthesia and revealed a large endotracheal tumor, blocking the airway almost completely (Fig. 1c). Bronchoscopic debulking was performed using electrocautery and cryotherapy, leaving a patent airway with a small residual tumor (Fig. 1d). The tumor was located 4 tracheal rings (approximately 2 cm) above the carina. Recovery was uneventful and the patient was discharged the next day without any remaining symptoms.

Histopathological examination showed an unclassifiable atypical myxoid spindle cell neoplasm with focal ALK expression and negative staining for keratins, EMA, TLE-1, p63, CD31, CD34, ERG, S100, SOX-10, TTF-1, SMA, desmin, myf4 and MUC4. Molecular analysis showed an EWSR1-CREB1 translocation, which can be found in primary pulmonary myxoid sarcoma (PPMS), AFH and in several other sarcomas. Under the working diagnosis of PPMS the patient underwent magnetic resonance imaging of both brain and kidneys and a whole body fluorodeoxyglucose positron emission tomography and CT. Both did not reveal any distant metastases.

The remaining tumor was removed through a cervical approach with a partial distal tracheal resection and end-to-end anastomosis with interrupted 4–0 PDS sutures (and two lateral interrupted 2–0 Vicryl sutures for approximation and anastomotic tension release) (Fig. 1e, patient’s head is left). The excised part of the trachea was cut open anteriorly and showed a tumor with a diameter of 15 mm located on the membranaceous portion (Fig. 1f). High-frequency jet ventilation was used to allow temporal surgical interruption of the trachea. The patient was extubated immediately after the procedure. Recovery was uneventful and the patient was discharged three days after surgery.

**Fig. 2:**
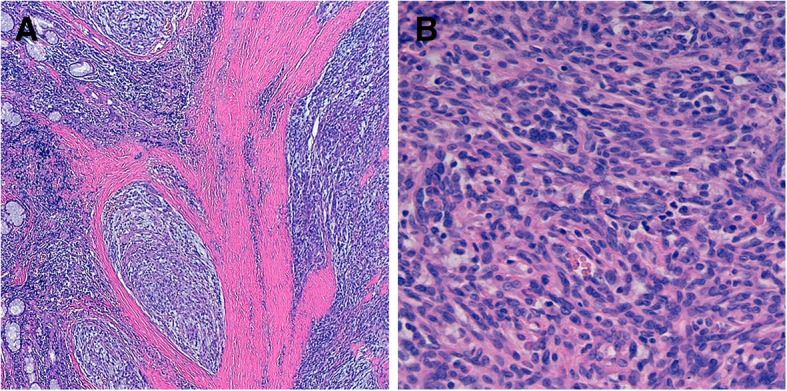
Histopathological examination of endotracheal angiomatoid fibrous histiocytoma

Microscopically, the tumor was removed completely. Histopathological examination at low power magnification showed distinct features of AFH with tumor nodules of variable size surrounded by a thick fibrous capsule with a rim of lymphoplasmacytic cells (Fig. 2a). High power magnification showed solid tumor nodules composed of bland myoid spindle cells (Fig. 2b). On follow-up, three years after surgery, the patient is asymptomatic, uses no asthma medication, has normal spirometry, and does not show any signs of recurrent tumor growth.

## Discussion and conclusions

AFH is a mesenchymal neoplasm of intermediate malignancy that generally affects children and young adults [1]. AFH occurs most commonly in the deep dermis or subcutis of extremities, followed by the trunk and head and neck [1]. AFH has a characteristic histological appearance simulating the appearance of a neoplasm occurring in a lymph node [1]. However, due to the variable histological appearance and the lack of consistently positive immunohistochemical markers, the diagnosis can be difficult [1]. Molecular genetic studies have shown three characteristic translocations and nearly 93% of AFH have a rearrangement of ESWR1 (often a EWSR1-CREB1 translocation), which is of diagnostic relevance [1]. However, the EWSR1-CREB1 translocation is also described in other tumors, such as PPMS [1, 4]. Although PPMS and AFH may represent an overlapping histologic spectrum, PPMS is consistently negative for desmin and characterized by a predominately reticular architecture and absence of a lymphoplasmacytic cuff [1, 4]. This case-report confirms the challenge in making a correct (histological) diagnosis of AFH, especially when it occurs at an unusual site.

There is one previous case report that describes endobronchial AFH in two cases [2] and only one case report that describes (upper) endotracheal AFH [3]. To our knowledge, surgical resection of endotracheal AFH has not been described before, nor has a hybrid bronchoscopic and surgical resection strategy. A staged resection was required in this case to quickly secure the airway, allowing a lower-risk planned surgical procedure for complete resection. We chose a cervical approach, instead of a more invasive, painful and cosmetically less satisfying thoracotomy. Although a thoracotomy is generally recommended for distal tracheal resections, we have shown that distal tracheal resection for endotracheal AFH can be safely performed in young adults through a cervical approach with excellent follow-up results.

## Abbreviations

AFH: angiomatoid fibrous histiocytoma
CT: computed tomography
PPMS: primary pulmonary myxoid sarcoma
